# Traffic Sign Detection and Quality Assessment Using YOLOv8 in Daytime and Nighttime Conditions

**DOI:** 10.3390/s25041027

**Published:** 2025-02-09

**Authors:** Ziyad N. Aldoski, Csaba Koren

**Affiliations:** 1 Department of Highway and Bridge, Technical College of Engineering, Duhok Polytechnic University, Duhok 1006, Kurdistan Region, Iraq; ziyad.nayef@dpu.edu.krd; 2 Department of Transport Infrastructure and Water Resources Engineering, Faculty of Architecture, Civil Engineering and Transportation Sciences, Széchenyi István University, 9026 Győr, Hungary

**Keywords:** traffic safety, traffic sign detection, YOLOv8 algorithm, retroreflectivity, autonomous vehicles, camera sensor, human evaluation

## Abstract

Traffic safety remains a pressing global concern, with traffic signs playing a vital role in regulating and guiding drivers. However, environmental factors like lighting and weather often compromise their visibility, impacting human drivers and autonomous vehicle (AV) systems. This study addresses critical traffic sign detection (TSD) and classification (TSC) gaps by leveraging the YOLOv8 algorithm to evaluate the detection accuracy and sign quality under diverse lighting conditions. The model achieved robust performance metrics across day and night scenarios using the novel ZND dataset, comprising 16,500 labeled images sourced from the GTSRB, GitHub repositories, and real-world own photographs. Complementary retroreflectivity assessments using handheld retroreflectometers revealed correlations between the material properties of the signs and their detection performance, emphasizing the importance of the retroreflective quality, especially under night-time conditions. Additionally, video analysis highlighted the influence of sharpness, brightness, and contrast on detection rates. Human evaluations further provided insights into subjective perceptions of visibility and their relationship with algorithmic detection, underscoring areas for potential improvement. The findings emphasize the need for using various assessment methods, advanced algorithms, enhanced sign materials, and regular maintenance to improve detection reliability and road safety. This research bridges the theoretical and practical aspects of TSD, offering recommendations that could advance AV systems and inform future traffic sign design and evaluation standards.

## 1. Introduction

Traffic safety remains a global challenge, with road crashes being among the leading causes of fatalities worldwide. Approximately 1.2 million deaths and 20 to 50 million non-fatal injuries occur annually due to road crashes [1]. Vulnerable road users, such as pedestrians and cyclists, account for a significant portion of these fatalities, with human error contributing to 93% of road crashes globally [2,3,4]. These alarming statistics highlight the urgent need for systems that minimize human error, such as Advanced Driver Assistance Systems (ADASs) and autonomous vehicle (AV) technologies. By automating critical driving tasks traditionally reliant on human decision-making, these systems hold significant potential to enhance road safety and reduce traffic-related incidents.

Among the key elements influencing road safety are traffic signs, which provide drivers with crucial regulatory, warning, and guidance information [5]. Failure to accurately detect or interpret these signs is a well-documented factor contributing to accidents, especially under challenging conditions such as poor lighting, adverse weather, or complex roadway environments [6,7]. Traffic sign detection (TSD) and classification (TSC) are inherently complex due to the wide variety of signs, each characterized by unique shapes, colors, and symbols, as well as their susceptibility to wear, damage, or environmental degradation [8]. This complexity is compounded in real-world scenarios where factors like visibility, weather, and lighting significantly impact the detection process.

Accurate TSD and TSC have become critical for ensuring that these systems operate safely and efficiently as AV technology advances. However, TSD is a challenging task, demanding substantial computational resources and a high degree of classification accuracy [9,10]. AVs must reliably detect and classify traffic signs across diverse environmental conditions, including variations in weather and lighting. Addressing this challenge requires developing and evaluating robust deep and machine learning models trained on comprehensive datasets such as the German Traffic Sign Recognition Benchmark (GTSRB) [11], which provides diverse traffic sign images suitable for training and validation purposes.

Despite considerable advancements in TSD, there are notable gaps in evaluating the quality of traffic signs and understanding how it impacts AV performance [12]. Existing research often prioritizes overall detection accuracy but does not adequately address the differentiation between high- and low-quality traffic signs. Additionally, the implications of varying traffic sign qualities on AV navigation systems remain underexplored. These gaps are especially critical in real-world scenarios, where factors such as poor lighting, adverse weather, or damaged traffic signs further compromise detection performance and AV navigation reliability [13,14].

This study addresses these gaps by developing and evaluating a new TSD and TSC model developed using the YOLOv8 (You Only Look Once version 8) algorithm. Specifically, the research explores the ability of camera-based systems to assess the quality of traffic signs under diverse lighting conditions, differentiates between high- and low-quality traffic signs, and investigates the implications of these factors on AV navigation. To achieve this, the study introduces a novel dataset called ZND comprising 16,500 meticulously labeled images sourced from the GTSRB, GitHub repositories, and photographs captured in Gyor, Hungary. The model is tested using video recordings collected during daytime and night-time to evaluate its performance in detecting and classifying traffic signs.

By analyzing the classification rates of individual traffic signs across these scenarios, this research provides actionable insights into how illumination factors affect detection accuracy. The findings contribute to enhancing AV perception systems by improving traffic sign recognition robustness and reliability. Ultimately, this study bridges the gap between theoretical advancements and practical applications, supporting the development of safer and more efficient AV technologies and influencing future traffic sign standards to improve global road safety.

## 2. Related Work

Accurately detecting and classifying traffic signs is a critical components of AV systems, and substantial research has addressed these challenges. Early methodologies relied on traditional image processing and classical machine learning techniques such as k-Nearest Neighbors (k-NN) [15] and Support Vector Machines (SVMs) [16]. While these methods were effective in structured and controlled environments, their performance deteriorated under dynamic and unpredictable conditions, such as variations in lighting, occlusions, and environmental distortions.

### 2.1. Detection Methods

The introduction of machine learning and deep learning marked a transformative shift in the field, with Convolutional Neural Networks (CNNs) emerging as the dominant approach due to their superior ability to extract features and generalize across varying conditions [17,18]. A key milestone in this evolution was the creation of the GTSRB dataset, which provided a standardized benchmark for training and evaluating models. Subsequent advancements led to the development of state-of-the-art architectures, such as YOLO (You Only Look Once), SSD (Single Shot MultiBox Detector), and Faster R-CNN, which excel in real-time detection and classification tasks [8,19]. For example, YOLOv3 incorporated a feature pyramid structure to enhance the detection of small objects, addressing a common challenge in traffic sign recognition. Variants like MSA_YOLOv3, which integrated multi-scale spatial pyramid pooling and data augmentation, demonstrated improved robustness under challenging environmental conditions [20,21].

Recent advancements in anchor-free architecture have further improved traffic sign detection. A notable example is a robust real-time anchor-free traffic sign detector utilizing a one-level feature architecture. This approach reduces false detection rates while maintaining high recall rates, highlighting the growing focus on adaptive algorithms that leverage multi-scale feature extraction and data augmentation to enhance performance [22].

Researchers have used data augmentation to mitigate these challenges, enriching training datasets with diverse environmental scenarios [23]. Multi-scale feature extraction techniques have also improved models’ adaptability to varying object sizes and shapes [24]. Additionally, hybrid architectures integrating spatial transformers with CNNs have shown promise in addressing occlusions and spatial distortions [25]. These approaches collectively enhance the resilience of traffic sign detection systems under real-world conditions [26]. These insights have driven the development of advanced methods, including data augmentation, to simulate diverse scenarios and hybrid models that combine spatial transformers with CNNs to address spatial invariance issues [27]. Training on diverse datasets, such as the Tsinghua-Tencent 100K, has further enhanced models’ robustness to environmental variability [21,28]

Innovations in multispectral detection systems, such as the Transformer Fusion-based Scale-aware Attention Network (TFSANet), have shown promise in addressing the challenges posed by low visibility and varying object sizes. By effectively integrating RGB and thermal images, TFSANet outperforms traditional methods, such as YOLOv5 and Faster R-CNN, under challenging environmental conditions, further emphasizing the potential of multispectral data in improving detection system robustness [29].

### 2.2. Traffic Sign Detection in Simulation vs. Real-Time

Simulation environments are pivotal in advancing traffic sign detection systems, offering controlled platforms for testing algorithms under diverse conditions. Tools like Gazebo, integrated with the Robot Operating System 2 (ROS 2), allow researchers to simulate various lighting, sensor noise, and environmental challenges [30]. These platforms facilitate algorithm validation while providing a foundation for practical, hands-on learning for AV engineers. Real-time implementations, in contrast, prioritize speed and adaptability, addressing real-world complexities such as occlusions, adverse weather, and dynamic lighting changes. YOLOF-F, for instance, utilizes single-level feature fusion for enhanced multi-scale detection in real-time scenarios, achieving superior performance even under challenging conditions [31].

### 2.3. Related Work in AV Applications

Accurate traffic sign detection is critical for AV systems, influencing navigation and safety. Environmental conditions, such as adverse weather and lighting inconsistencies, significantly impact detection performance [13,21]. For instance, rain, fog, and nighttime glare increase false-negative rates, highlighting the need for algorithms that adapt to such variability [32]. Studies have also explored the interplay between road design and sensor placement. Khaska and Miletics (2023) demonstrated that outdated road guidelines for driver’s eye height fail to account for AV sensor advancements, emphasizing the importance of aligning infrastructure design with evolving AV technologies [33,34].

### 2.4. Traffic Sign Quality and Retroreflectivity

Traffic sign retroreflectivity is vital for visibility under low-light and night-time conditions. Retroreflective surfaces degrade over time due to weathering, pollution, and physical damage, compromising safety and detection accuracy [5,35,36,37,38,39,40,41,42,43]. Aldoski et al. (2024) [44] highlighted the role of retroreflectivity assessments in linking reduced retroreflectivity to decreased detection rates in low-light environments. Advanced tools, such as handheld retroreflectometers and automated systems, facilitate precise evaluations, ensuring compliance with safety standards and timely maintenance [5].

Integrating retroreflectivity data with real-world video analysis offers a novel perspective on enhancing traffic sign detection. This dual approach addresses algorithmic robustness and physical sign degradation, providing a comprehensive framework for developing resilient detection systems [13]. By combining insights from retroreflectivity assessments and dynamic video analysis, researchers can optimize algorithms to account for environmental and physical factors, advancing AV safety and reliability.

This review underscores the advancements in traffic sign detection and classification, emphasizing the importance of evaluating system performance across diverse real-world scenarios. Integrating video-based evaluations with retroreflectivity assessments offers a comprehensive framework for developing robust and effective detection systems, ensuring their reliability in AV technologies and beyond.

## 3. Methodology

This methodology outlines the data collection and analysis procedures leveraging handheld retroreflectometers and AV cameras to assess the quality of traffic signs. By integrating these tools, the framework offers a systematic approach to ensuring accurate, reliable data acquisition and examination. The results can be further utilized for detailed analysis, driving traffic signal evaluation and management improvements.

### 3.1. Study Area

**Figure 1:**
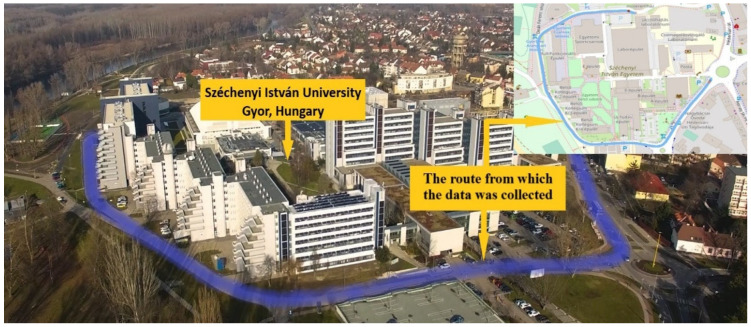
Study location [44].

The investigation conducted in this study took place on the Széchenyi István University campus, situated in Győr, Hungary, as depicted in Figure 1. The campus features diverse traffic signs, providing an ideal setting for data collection using handheld devices and camera technology. The test track was fully equipped with street lighting along its route.

### 3.2. Data Collection

#### 3.2.1. Creating the ZND Dataset

**Figure 2:**
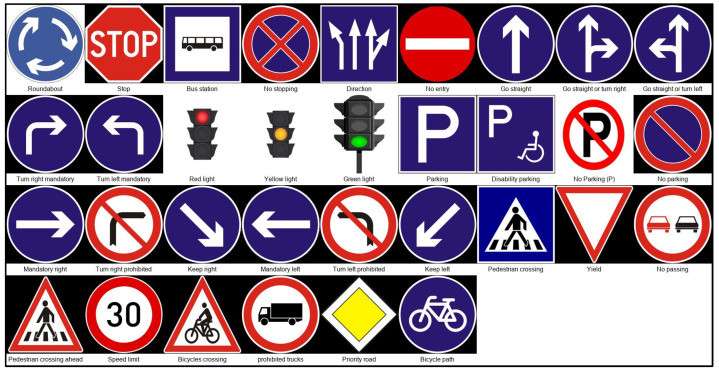
Images and class labels within the ZND database.

**Figure 3:**
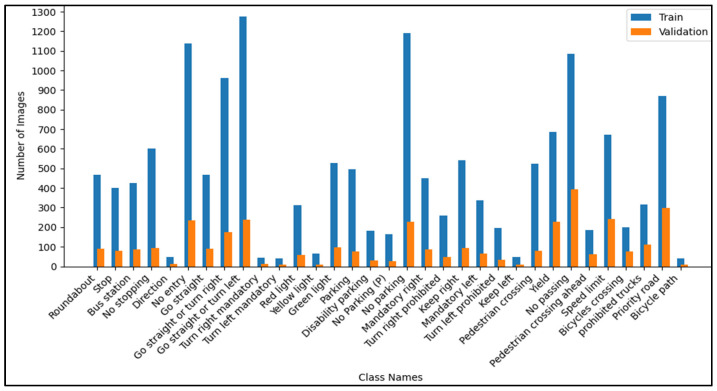
Class distribution in training and validation sets of the ZND database.

The ZND dataset, used for training the model, consists of 16,500 labeled images of traffic signs categorized into 33 distinct classes, as shown in Figure 2. These classes include various regulatory, warning, and guidance signs, such as Stop, No Entry, Pedestrian Crossing, Speed Limit, and Priority Road, as illustrated in Figure 3. Each image in the dataset was meticulously labeled using the LabelImg tool, which allowed for the precise annotation of traffic signs within the pictures, including bounding boxes and class labels. Various platforms contributed to the dataset, including the GTSRB, GitHub repositories, and real-world photographs taken in Gyor, Hungary. The images in the dataset were carefully curated to cover different environmental and illumination conditions and capture a wide variety of angles, lighting, and backgrounds.

The dataset was structured as follows:

- **Training set:** 80% of the images were used for training the YOLOv8 model, ensuring the diverse representation of all 33 classes.
- **Validation set:** The remaining 20% of the images were used for validation to evaluate the model’s performance on unseen data.

#### 3.2.2. Video Recording

Video recordings were conducted to evaluate the model’s performance in practical, real-world conditions. These recordings were performed in the study area with diverse traffic signs. Videos were recorded under different lighting conditions (daytime and nighttime) to simulate real-world scenarios.

**Figure 4:**
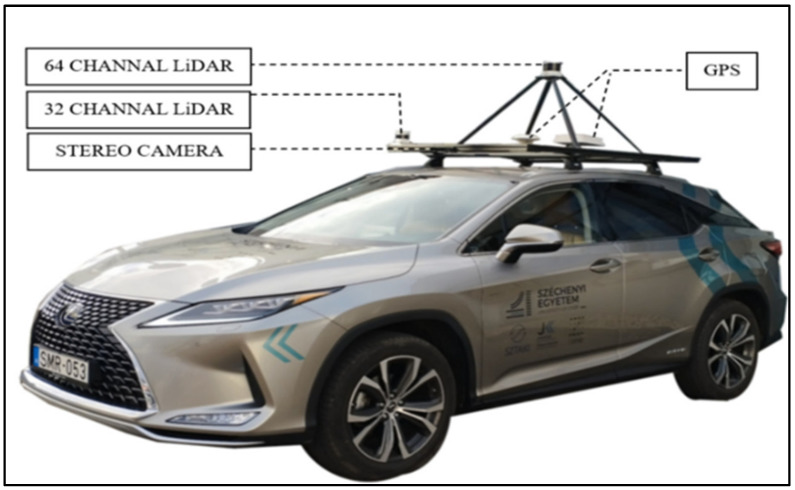
Lexus RX450h vehicle fitted with sensors, adapted from [45].

The high-resolution videos from the AV Stereolabs ZED2i camera, as shown in Figure 4, provided diverse perspectives on the traffic signs. These videos assessed the model’s performance in detecting and classifying traffic signs in varying conditions and examining their visibility and quality, ensuring a comprehensive evaluation of the model’s robustness in real-world applications.

#### 3.2.3. Handheld Retroreflectometer Data

In addition to the video data, retroreflectivity measurements were collected using the RetroSign GRX 554 handheld retroreflectometer owned by the Road Laboratory of the University. DELTA manufactured the equipment as a part of FORCE Technology (Hørsholm, Denmark), which determines the coefficient of retroreflection (RA) for traffic signs. This device complies with the European standard EN 12899-1 [46], and provides measurements for illumination angles of 5° and observation angles of 0.2°, 0.33°, and 1°. Among these, an observation angle of 0.33° was used as the reference for the retroreflectivity measurements of traffic signs.

The measurement process involved the following:

- Calibration: The handheld retroreflectometer was calibrated by placing the calibration standard on the device’s calibration side.
- Measurement: The device was positioned perpendicular to the traffic sign surface, and measurements were taken for the background and legend of the traffic sign. Four readings were collected for each sign to ensure accuracy.

**Figure 5:**
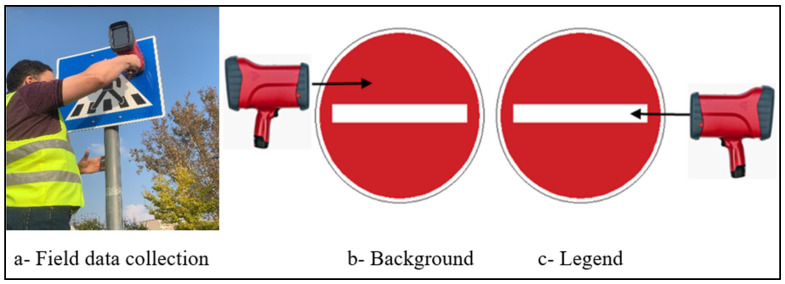
RA measurement of sign background and legend.

The retroreflectivity coefficient values used in this study represent the average of the measurements for the two primary components of the traffic signs (background and legend). This methodology was chosen to align with the functionality of the camera-based detection system, which processes and utilizes the entire visible color spectrum present on the traffic signs. By using this approach, the study accounts for the combined retroreflectivity of all colors on the sign, thus reflecting the real-world detection conditions where multiple colors influence the camera’s performance. These retroreflectivity measurements were subsequently used for a comparison with the results of the camera-based detection system and evaluate the correlation between physical retroreflectivity data and the camera’s detection performance. Figure 5 illustrates the process of field data collection.

#### 3.2.4. Human Evaluation

The human evaluation of traffic sign visibility was conducted using two complementary methods: on-site assessment and an online survey. Both methods employed a standardized questionnaire featuring three key questions about traffic sign visibility, readability, and contrast (Table 1). The results from both methods were averaged due to the strong correlation between the three questions, ensuring a robust comparison of the findings.

##### On-Site Evaluation

**Figure 6:**
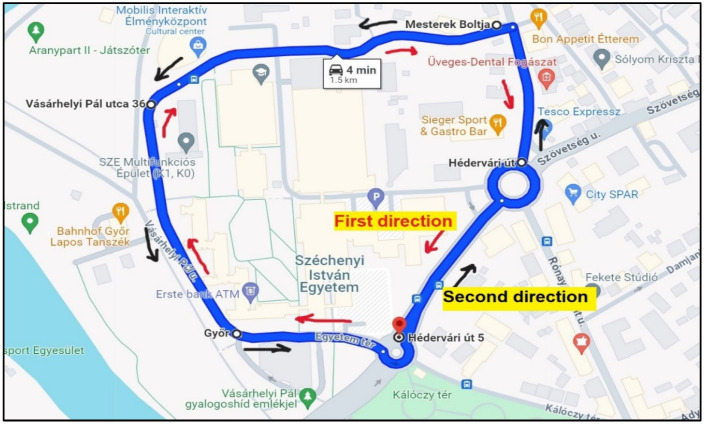
Illustration of the study area and data collection route.

The methodology for the on-site human evaluation of traffic sign visibility was designed to ensure a systematic framework for data collection. The process followed a sequence of steps outlined as follows:

- Survey design: A survey with three questions tailored to evaluate the visibility of each traffic sign (Table 1) was created. An illustrative map was also provided to guide the evaluators and aid in identifying the location of the signs, as shown in Figure 6. The human evaluation of traffic signs was conducted exclusively during daytime conditions.
- Traffic sign identification: Each traffic sign was assigned a unique identification number clearly marked on the supporting pole to facilitate easy identification during the evaluation. In cases where multiple signs were mounted on the same pole, sequential numbering was used from top to bottom to ensure a clear and consistent method of sign identification.
- Volunteer safety and preparation: All volunteers participating in the survey were equipped with safety vests to ensure their visibility and safety during the evaluation process. This measure minimized any risks associated with their presence on-site. Additionally, the evaluation was conducted while walking rather than driving, ensuring a more detailed and accurate assessment of each traffic sign’s visibility.
- Survey administration: The survey was administered individually to each volunteer, allowing them to assess the traffic signs. This individualized approach ensured that each volunteer’s evaluation was captured without influence from others.

##### Online Survey

An online survey was conducted to complement the on-site evaluation. This survey utilized the same questionnaire as the on-site evaluation, featuring three questions about traffic sign visibility, readability, and contrast (Table 1). The survey was administered to 61 participants, who evaluated 40 images of traffic signs captured in the study area.

To ensure consistency in comparison, the average of the three questions was used for each participant’s responses, mirroring the approach taken in the on-site evaluation. This approach allowed for a systematic comparison between the on-site and online evaluation results, leveraging the strong correlation between the three questions to enhance the reliability of the findings.

After data collection using the human evaluation methods, the responses from both the on-site and online evaluations were carefully organized and stored in an Excel spreadsheet to facilitate further analysis. The scores for the three questions, which exhibited a strong correlation, were averaged to create a single representative score for each participant. These average scores were divided by 5 to standardize the evaluation metrics, converting them to a 0–1 scale consistent with the camera-based detection rate scale. This standardized approach ensured alignment across different evaluation methods and enhanced the comparability and reliability of the findings.

### 3.3. Model Development

#### 3.3.1. YOLOv8 Model Training

**Figure 7:**
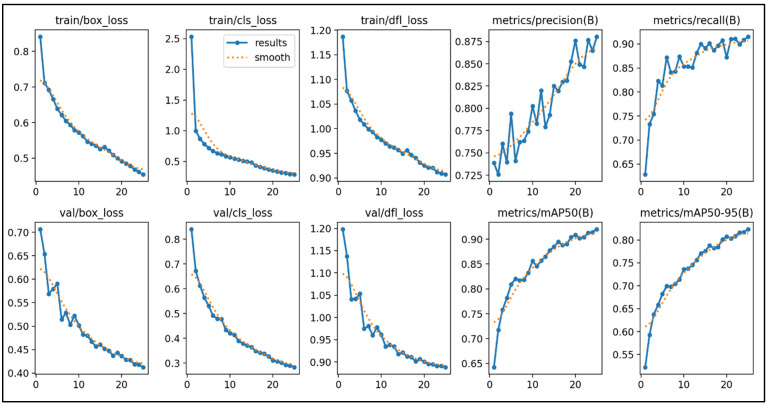
Training YOLOv8 performance metrics.

Due to its efficiency and real-time performance, the traffic sign detection and classification model was developed using the YOLOv8 algorithm, specifically the YOLOv8s architecture. The training process involved the following:

- Dataset preparation: The ZND dataset, with 16,500 labeled images from 33 traffic sign classes, was used to train the model. This extensive dataset provided diverse traffic sign images, ensuring that the model could generalize to various real-world scenarios.
- Model configuration: The YOLOv8 model was configured with optimized parameters to ensure a robust performance, including an input image size of 640 × 640, a learning rate of 0.01, and a batch size of 16. The process was carried out in a high-performance computing environment on Kaggle, leveraging the NVIDIA A100 GPU for accelerated computations and efficient convergence. Specifically, the model was trained over 50 epochs, with the learning rate and other hyperparameters carefully tuned to achieve the best results.
- Training process: The model underwent training over multiple epochs to minimize the loss function and achieve high accuracy. The training process involved the following key metrics and observations:
  - Training losses: Throughout 50 epochs, the training box loss decreased from 0.85 to 0.41, the classification loss reduced from 2.5 to 0.2, and the DFL loss dropped from 1.2 to 0.8. These reductions indicate that the model effectively learned to detect and classify traffic signs with increasing precision.
  - Validation losses: The validation losses also showed significant improvement, with the box loss decreasing from 0.71 to 0.39, the classification loss reducing from 0.85 to 0.22, and the DFL loss dropping from 1.2 to 0.8. These metrics demonstrate the model’s ability to generalize well to unseen data.
  - Performance metrics: The final precision bounding box (B) reached 0.88, the recall (B) was 0.92, mAP50 (B) was 0.92, and mAP50-95 (B) was 0.82. These high values indicate that the model achieved excellent performance in detecting and classifying traffic signs across different Intersections over Union (IoU) thresholds. Figure 7 shows the performance metrics.

The YOLOv8 model demonstrated a robust traffic sign detection and classification performance, with high precision, recall, and mean Average Precision (mAP) metrics. The training process, supported by a comprehensive dataset and high-performance computing resources, ensured that the model could effectively handle variations in sign size, position, and environmental factors.

##### Data Augmentation Techniques

Several data augmentation techniques were employed to enhance the diversity and robustness of the training data for the YOLOv8 model. These augmentations were designed to simulate real-world variations in traffic sign appearances, ensuring the model’s generalization ability across diverse scenarios.

Dataset Preparation:
  - Images were sourced from multiple datasets, including the GTSRB, GitHub repositories, and photographs captured using a high-resolution camera.
  - Additional images were extracted from video recordings using Python-based processing methods, ensuring the inclusion of varied environmental and illumination conditions.
Augmentation Methods:
  1. Cropping:
    - Traffic signs were cropped from larger images to focus on their specific regions of interest. This technique helped train the model to accurately detect signs, even when embedded in cluttered backgrounds.
  2. Synthetic Image Generation:
    - Traffic signs were inserted into other background images to simulate real-world scenarios where signs appear in various contexts. These synthetic images included the following:
    - Different Sizes: Traffic signs were resized to represent varying distances from the camera.
    - Rotations: Signs were rotated to replicate angles commonly observed in real-world conditions, such as tilted or slightly skewed signs.

This approach introduced additional variability into the dataset by merging traffic signs with diverse backgrounds, reflecting the complexity of real-world conditions.

#### 3.3.2. Model Testing and Evaluation

Once trained, the YOLOv8 model was tested on categorized video data recorded under varying lighting conditions. The goal was to assess how well the model could detect and classify traffic signs under real-world conditions. As shown in Figure 7, the evaluation metrics included detection rates, the classification accuracy, and the quality of the detected signs based on factors like sharpness, brightness, and contrast.

### 3.4. Evaluation Metrics

Several key metrics were extracted and analyzed to assess the performance of the YOLOv8 model and evaluate the visibility and quality of traffic signs under varying lighting conditions. These metrics are categorized into detection metrics and sign quality metrics, as detailed below:

#### 3.4.1. Detection Metrics

The detection metrics used in this evaluation were designed to measure the accuracy and performance of the YOLOv8 model in detecting traffic signs. The following metrics were computed:

- **Precision:** This metric measures the proportion of correctly identified traffic signs among all detected instances, indicating the accuracy of the detection model in terms of false positives.
- **Recall:** Recall assesses the proportion of actual traffic signs correctly detected by the model, reflecting the model’s ability to identify all relevant signs within the input data.
- **F1-Score:** The F1-Score represents the harmonic mean of precision and recall, providing a balanced measure of the detection model’s accuracy and completeness.
- **Detection rate:** The detection rate refers to the maximum percentage of traffic signs correctly identified and classified by the detection model (YOLOv8), a deep learning (DL) model, under specific conditions. This metric represents the highest percentage of traffic signs detected across all video frames. With video recordings captured at 20 frames per second, the peak detection performance observed across all frames determines the detection rate.

#### 3.4.2. Sign Quality Metrics

In addition to the detection metrics, the following visibility and quality metrics were computed for each detected traffic sign to assess their clarity and overall quality:

- **Overall intensity:** The average pixel intensity of the entire sign reflects the traffic sign’s brightness and contrast.
- **Sharpness:** This was measured using Laplacian variance, indicating the clarity of the sign and its edges.

These metrics were extracted from the detected traffic signs within video frames and used to evaluate the effects of lighting conditions (day vs. night) on the visibility and detectability of traffic signs. This detailed analysis allowed for a comprehensive assessment of the traffic sign quality and detection performance under diverse real-world conditions.

## 4. Results and Discussion

This section presents a deep analysis of the traffic sign detection and classification performance of the YOLOv8 model under various light conditions. This section delves into the detection rates, classification accuracy, and quality metrics of traffic signs, highlighting the impact of lighting on the model’s effectiveness. By examining these results, the study provides valuable insights into the robustness and reliability of the model in real-world scenarios, ultimately contributing to the enhancement of AV perception systems.

### 4.1. Detection Rate Comparison

The detection rate refers to the maximum percentage of traffic signs correctly identified and classified by a detection model (YOLOv8) within a specific set of conditions. This metric evaluates the model’s peak performance in accurately detecting traffic signs. The detection rate of traffic signs by AV systems varies significantly between day and night due to differences in lighting conditions. During the day, traffic signs are generally more visible, resulting in a higher detection rate. Natural lighting enhances the clarity and contrast of the signs. In contrast, night-time conditions pose challenges such as reduced illumination, glare from external light sources, and partial occlusion, which can obscure sign details and reduce detection accuracy.

Table 2 presents the distribution of detection rates between day and night, highlighting the model’s ability to perform consistently under varying lighting conditions. The detection rate during the day shows a mean of 0.82 with a relative standard deviation (RSD) of 22%, while the night detection rate has a slightly higher mean of 0.83 and a lower RSD of 20%. This minor variation implies that the model performs reliably under both conditions.

An independent two-sample *t*-test was conducted to examine whether there is a significant difference in the mean detection rate between day and night. The analysis, presented in Table 3, was performed using the Statistical Package for the Social Sciences (SPSS) software, version 27.0.1, with a significance level (α) of 0.05, a two-tailed test, and 178 degrees of freedom. The critical value (CV) from the student’s t-distribution was calculated to be 1.973, and the resulting *t*-value of −0.34 is lower than the critical value. The corresponding *p*-value of 0.736 exceeds the chosen significance level, leading to the acceptance of the null hypothesis, which was that there is no statistically significant difference in the mean detection rates between daytime and night-time. The 95% confidence interval for the difference in means includes zero, further reinforcing this conclusion.

**Figure 8:**
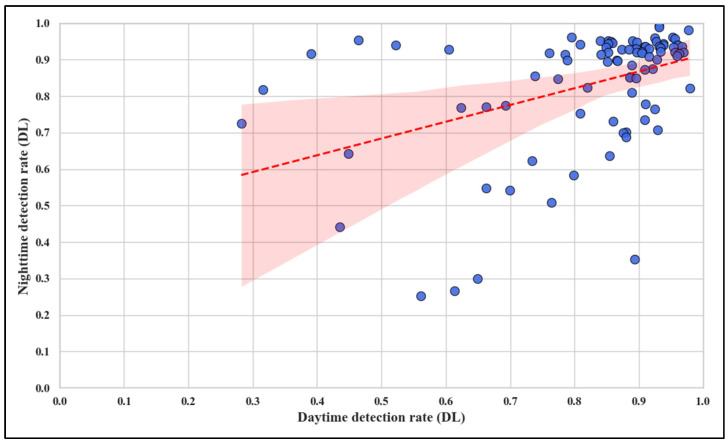
Comparison of detection rate: daytime vs. night-time.

However, while the mean detection rates and standard deviations show no significant overall difference, a detailed individual analysis reveals noteworthy variability. Figure 8 investigates the relationship between daytime and night-time detection rates, showing only a weak positive correlation, with Pearson and Spearman coefficients of 0.42 and 0.44, respectively. The weak correlation indicates that although signs with higher daytime detection rates are likely to perform well at night, the relationship is not definitive. Notably, as shown by the outliers in Figure 8, signs that deviate markedly from the trend line highlight potential factors such as reduced retroreflectivity, glare, shadows, or environmental occlusions, particularly at night. Additionally, signs with consistently low detection rates across both conditions may reflect poor quality, degradation, or suboptimal positioning, necessitating targeted investigation and maintenance.

**Figure 9:**
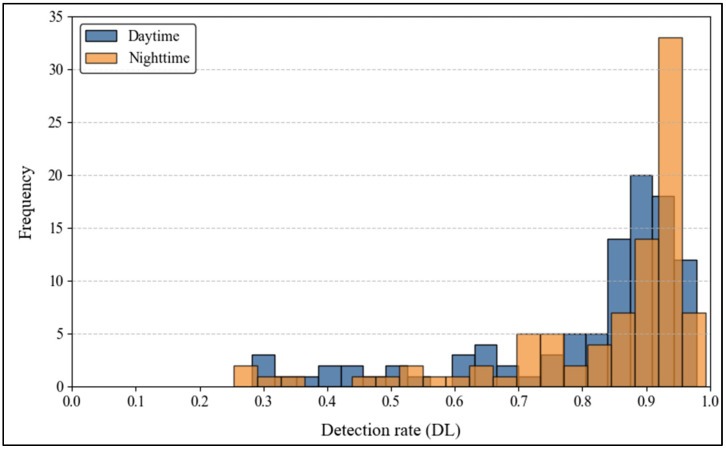
Distribution of daytime vs. night-time detection rate.

Figure 9 provides a comparative distribution of daytime and night-time detection rates. The data show a mean detection rate of 0.82 during the day and 0.83 at night, with relative standard deviations (RSDs) of 22% and 20%, respectively. While the distributions appear similar overall, the higher frequency of misdetections at night suggests challenges such as glare and reduced illumination. The model performs consistently across both conditions on average, but the broader spread of night-time data indicates variability in detection performance. Understanding the factors contributing to this variability, such as poor sign retroreflectivity or challenging lighting conditions, is essential for improving detection algorithms, particularly under night-time conditions.

**Figure 10:**
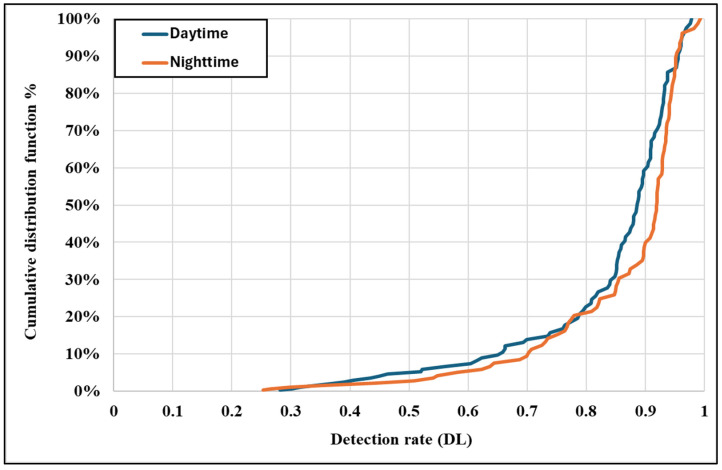
Cumulative distribution function (daytime and night-time).

Figure 10 illustrates the cumulative distribution functions (CDFs) of detection rates for daytime and night-time conditions. The CDFs reveal that night-time conditions exhibit a steeper cumulative curve than daytime, indicating that a greater proportion of signs are detected at lower rates at night. The lower tail of the night-time CDF suggests that specific traffic signs experience a significantly reduced detection performance under low-light conditions. These discrepancies emphasize the critical impact of illumination and glare on detection capabilities. Detection systems could achieve a consistent performance across varying lighting conditions by integrating adaptive exposure control and low-light image enhancement, particularly at night.

**Figure 11:**
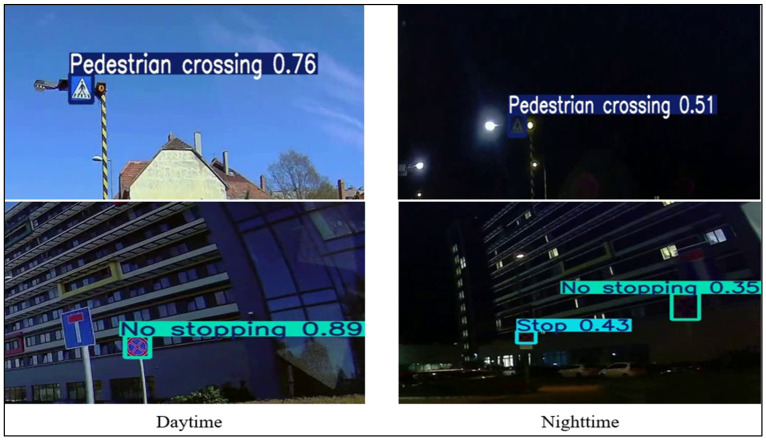
Detection rate variability for traffic signs under daytime and night-time conditions.

Furthermore, Figure 11 visually illustrates the detection rates for the same traffic signs under daytime and night-time conditions. The figure emphasizes that traffic signs are generally easier to detect during the day due to the better clarity and contrast provided by natural lighting. At night, however, the detection performance is heavily dependent on the retroreflective properties of the signs. Well-maintained retroreflective signs are more visible, while those with degraded retroreflectivity or affected by glare and shadows are often missed or misclassified. These findings underscore the importance of assessing and maintaining retroreflectivity and the need for regular retroreflectivity evaluations to ensure a consistent detection performance, particularly under challenging night-time conditions. Moreover, glare-reducing and adaptive imaging algorithms can significantly enhance the reliability of traffic sign detection systems in autonomous vehicles.

**Figure 12:**
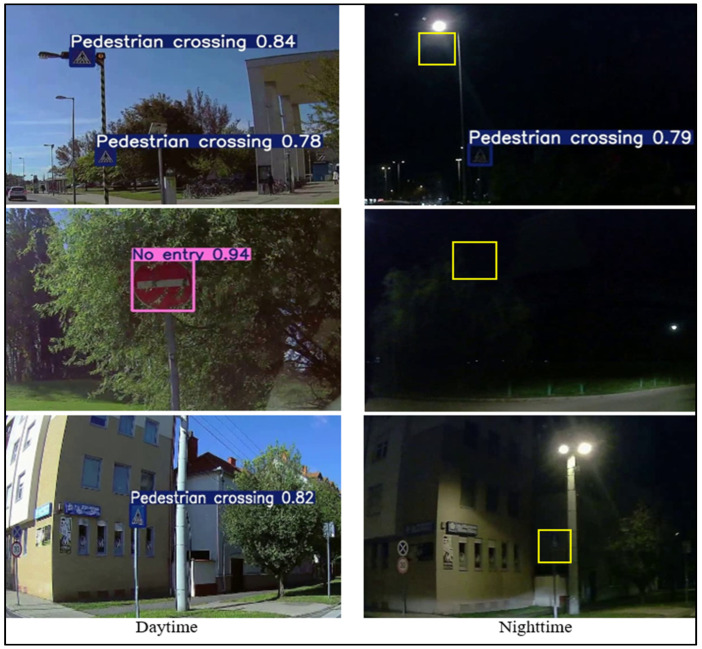
Examples of traffic signs detected during daytime but missed at night-time.

The data reveal that 10% of traffic signs were missed during night-time detection despite being detected during the day. This discrepancy highlights the specific challenges associated with night-time detection, particularly under low-light conditions. For instance, one of the signs that was detected during the day may have had sufficient visibility due to good natural lighting. Still, it was missed during the night, possibly because of degraded retroreflectivity or obstructions like glare from streetlights or nearby vehicle headlights. These signs are difficult to detect under night-time conditions when environmental factors affect the clarity of the signs. Figure 12 shows examples of such traffic signs: the same sign being detected during the day with a high level of clarity and contrast and the same sign failing to be detected at night due to the reduced retroreflectivity and glare.

The night-time detection performance is significantly affected by environmental factors that influence traffic sign visibility. While streetlights are intended to improve illumination, they can create uneven lighting, glare, or overexposure, impairing detection algorithms’ ability to identify fine details. Similarly, glare from oncoming headlights and reflective surfaces can obscure sign edges or symbols, reducing the effectiveness of camera-based detection systems. Shadows cast by infrastructure or moving vehicles further distort visibility, complicating detection. These challenges highlight the need for adaptive exposure controls and glare reduction techniques in detection algorithms. Integrating real-time lighting adjustments and regular retroreflectivity assessments can enhance low-light detection, particularly for autonomous systems in varied night-time conditions.

The results align with previous research highlighting the critical role of environmental conditions in traffic sign detection. Seraj et al., (2021) [13] emphasized the detrimental effects of low visibility, glare, and occlusions on detection accuracy. Similarly, other studies have shown that night-time conditions exacerbate challenges for AV systems, including increased false-negative rates and misclassifications [32]. The steeper cumulative distribution curve for night-time detection rates (Figure 10) supports these findings, indicating that a higher proportion of signs are detected at lower rates under low-light conditions.

While prior studies using advanced preprocessing techniques and hybrid architectures report improved robustness in night-time detection, the YOLOv8 model appears more sensitive to environmental variability. This is evidenced by the weak correlation in detection rates between day and night and the presence of outliers (Figure 8). These findings suggest that while YOLOv8 achieves commendable mean detection rates, its performance is less consistent under challenging conditions compared to models employing multispectral imaging, adaptive exposure control, or glare reduction algorithms [13,32].

The visual analysis presented in Figure 11 highlights the critical role of retroreflectivity in night-time detection. Signs with well-maintained retroreflective properties exhibit higher nighttime detection rates, while those with degraded retroreflectivity or obstructed by glare and shadows are more prone to misdetections. These results underscore the need for regular maintenance and retroreflectivity evaluations, as also advocated in the related literature.

### 4.2. Impact of Retroreflectivity on Detection Performance

Retroreflectivity plays a critical role in ensuring the visibility and detectability of traffic signs, particularly under varying light conditions. A higher retroreflectivity coefficient (Ra) improves the ability of drivers and detection systems to recognize traffic signs effectively, especially during night-time when artificial illumination is necessary for reflection. Conversely, lower retroreflectivity can result in an inconsistent or poor detection performance due to inadequate light reflection. The retroreflective properties of a sign are highly dependent on the material used, commonly categorized into classes such as RA1 and RA2. Signs with higher retroreflectivity values (e.g., RA2) demonstrate superior detection rates in daytime and night-time conditions than signs with lower retroreflectivity (e.g., RA1). It is important to note that retroreflective sheet class RA3 was not included in the scope of this study.

**Figure 13:**
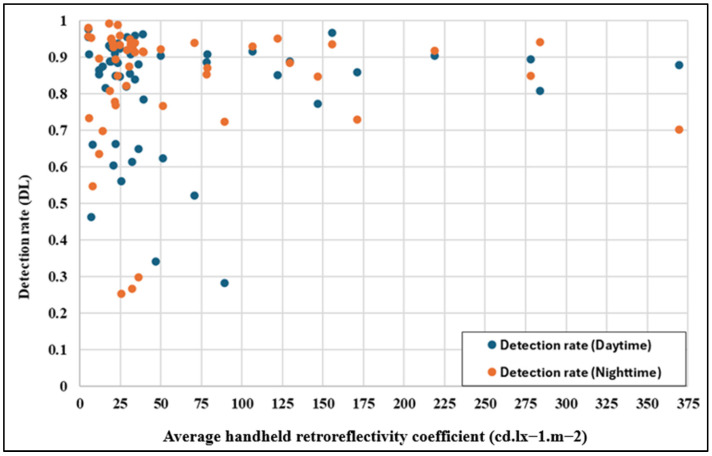
Relationship between retroreflectivity and the detection rate.

Figure 13 illustrates the relationship between retroreflectivity (Ra) and the detection rate under daytime and night-time conditions for two different classes of materials, RA1 and RA2. When the retroreflectivity exceeds 100 cd. lx^−1^.m^−2^, the detection performance remains consistently high for both light conditions. This trend highlights the effectiveness of materials with higher retroreflectivity in providing reliable detection rates. Conversely, when the retroreflectivity falls below 100 cd. lx^−1^.m^−2^, as seen with RA1, there is a noticeable variation in detection performance, particularly at night-time. The significant drop in detection consistency can be attributed to the limited light reflection of these lower-grade materials, which affects night-time visibility more severely than daytime performance.

Table 4 provides a detailed statistical comparison of the retroreflectivity and detection performance for RA1 and RA2 materials.

- RA1 signs exhibit low retroreflectivity, with a mean Ra of 29 cd. lx^−1^.m^−2^, a standard deviation (STD) of 18.0 and a relative standard deviation (RSD) of 63%, indicating significant variability in their reflective properties. This inconsistency in retroreflectivity directly impacts detection rates. The detection rate during the day has a mean of 0.8, with an STD of 0.17 and an RSD of 21%, reflecting moderate variability. At night, the mean detection rate is slightly higher at 0.83, but with more significant variability (STD = 0.19, RSD = 23%). Therefore, RA1 materials, with their lower retroreflectivity, are particularly prone to inconsistent detection performance under night-time conditions when retroreflection is critical.
- In contrast, RA2 signs exhibit significantly higher retroreflectivity, with a mean Ra of 187 cd. lx^−1^.m^−2^, an STD of 89.9 and an RSD of 48%, demonstrating better reflective properties despite moderate variability. The detection rate during the day for RA2 is 0.88, with a very low STD of 0.06 and an RSD of 6%, indicating remarkable stability in daytime performance. RA2 signs maintain excellent performance at night-time, with a mean detection rate of 0.87, an STD of 0.08, and an RSD of 9%. This stability highlights the direct correlation between the higher retroreflectivity and consistent detection performance across varying light conditions.

The findings from Table 4 reinforce the observation in Figure 13: RA2 materials, with significantly higher retroreflectivity, outperform RA1 materials by delivering stable and reliable detection rates during daytime and night-time conditions. In contrast, RA1 materials show substantial variability, particularly at night, due to their insufficient retroreflective properties.

**Figure 14:**
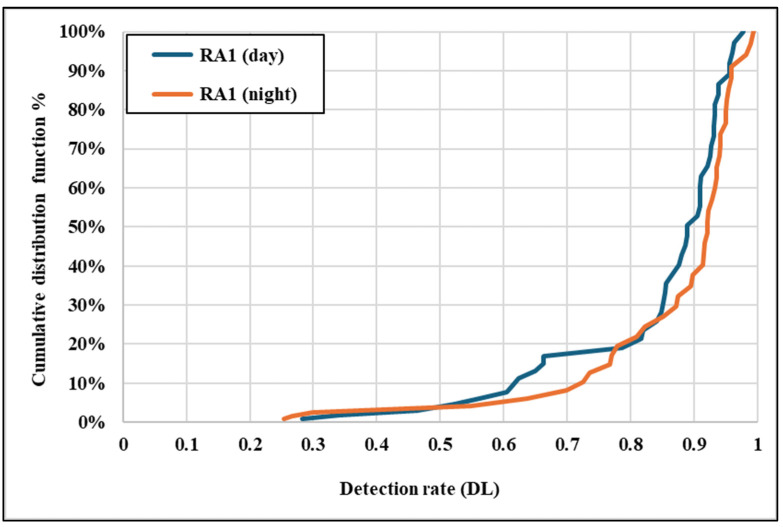
CDF of detection rates for RA1 class signs.

Figure 14 presents the CDF for RA1 detection rates under daytime and night-time conditions. The daytime CDF shows a gradual slope, with detection rates steadily increasing and reaching values close to 0.98. This indicates that RA1 traffic signs generally achieve moderate daily detection rates despite their lower retroreflectivity (mean Ra = 29). However, the night-time CDF exhibits a steeper slope, with detection rates starting much lower (around 0.25) and displaying significant variability. The slower growth and broader spread of detection rates reflect the difficulty of detecting RA1 signs under low-light conditions due to an inadequate retroreflective performance. This highlights the limitations of RA1 materials, where low Ra values result in a reduced and less reliable detection performance, particularly at night.

**Figure 15:**
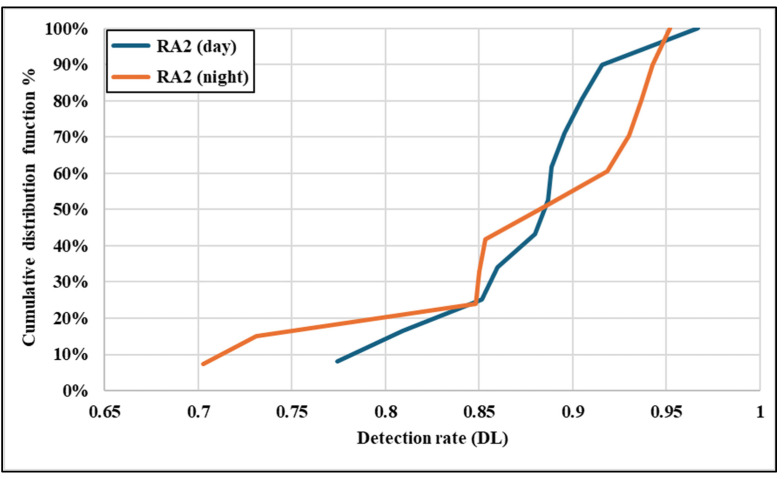
CDF of detection rates for RA2 class signs.

Figure 15 illustrates the CDF for RA2 detection rates under different lighting conditions. The daytime CDF shows a steep slope, with detection rates consistently above 0.77 and reaching up to 0.97, reflecting the excellent performance of RA2 signs during daylight. The clustering of detection rates at the higher end indicates minimal variability, supported by the low standard deviation and RSD values for the daytime performance of RA2. Similarly, the night-time CDF for RA2 starts at a relatively high value (0.70) and quickly rises toward the maximum (0.95), demonstrating strong performance even under low-light conditions. The consistency of the night-time detection rates is attributed to the higher retroreflectivity (mean Ra = 187), which enables superior light reflection and ensures high visibility. Compared to RA1, RA2 signs exhibit significantly less variability in both conditions, as indicated by the uniform slope of the CDF curves.

The data presented in Figure 13, Figure 14 and Figure 15 and Table 4 demonstrate the critical role of retroreflectivity in traffic sign detection performance. RA2 materials with higher retroreflectivity values exhibit consistent and superior detection rates under both daytime and night-time conditions. In contrast, RA1 materials, with lower retroreflectivity and higher variability, show reduced and inconsistent performance, particularly at night. These findings emphasize the importance of maintaining sufficient retroreflectivity in traffic signs, mainly through higher-grade materials like RA2, to ensure an optimal detection performance and road safety under all lighting conditions.

This study’s findings align with prior research emphasizing the critical role of retroreflectivity in traffic sign detection, particularly under night-time and low-light conditions. Higher retroreflectivity materials (e.g., RA2) demonstrated superior and a consistent detection performance compared to lower-grade materials (RA1), mirroring the trends reported by Aldoski et al., (2024) [44] and Seraj et al., (2021) [13]. The observed variability in RA1 detection rates under night-time conditions reinforces the limitations of lower retroreflectivity materials, as previously noted in studies addressing environmental and lighting challenges [13,23]. These results complement advancements in detection algorithms, such as YOLOv3 and TFSANet, which have shown improved robustness under dynamic conditions [20,23]. Furthermore, this study highlights the importance of integrating retroreflectivity assessments into autonomous vehicle (AV) detection systems, supporting findings that physical material properties significantly impact detection accuracy [5,32]. These comparisons underscore the necessity of higher-grade retroreflective materials to enhance detection reliability in real-world scenarios.

### 4.3. Overall Intensity

The overall intensity refers to the average pixel intensity of an entire traffic sign in an image, serving as a crucial metric for evaluating a sign’s brightness, contrast, and visibility. This parameter is critical for human drivers and automated detection systems, as higher intensity values are associated with improved detectability. The intensity is determined by averaging the pixel values across the traffic sign’s area, where in an 8-bit grayscale image, the pixel values range from 0 (black) to 255 (white). The visibility and detectability of traffic signs depend heavily on external factors such as the lighting conditions, retroreflective properties, and the quality of materials. A direct comparison between daytime and night-time intensities reveals significant disparities, which influence detection performance under varying light conditions.

The statistical analysis clearly contrasts the daytime and night-time overall intensity values, as shown in Table 5. During the daytime, the mean overall intensity is 128, with an STD of 52 and an RSD of 41%, indicating a moderate variation in intensity across traffic signs. This variability can be attributed to ambient lighting, sign positioning, and material retroreflectivity differences. In comparison, the night-time overall intensity exhibits significantly lower values, with a mean of 42, an STD of 19, and an RSD of 45%. The higher relative variability during night-time suggests that certain traffic signs struggle to reflect sufficient light, reducing brightness. This can be attributed to lower retroreflectivity in some signs or environmental challenges, such as glare, shadows, or occlusions. The reduced mean intensity at night highlights the critical role of retroreflective materials, as visibility in low-light conditions relies entirely on the reflection of artificial illumination.

**Figure 16:**
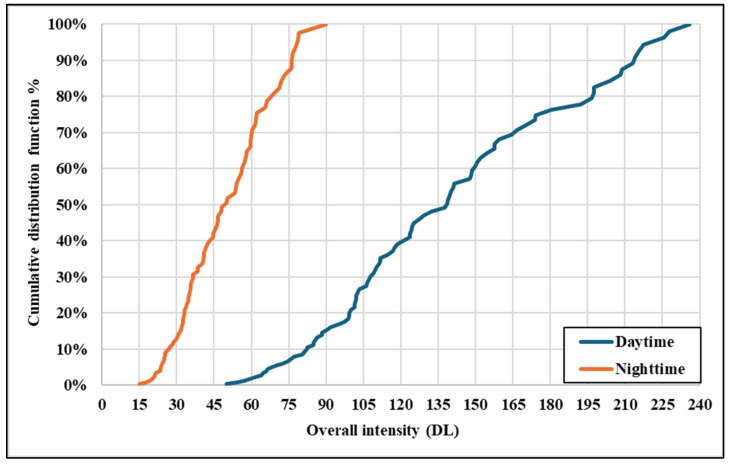
CDF for overall intensity between daytime and night-time.

The daytime CDF demonstrates a wider spread of intensity values, with a gradual slope and clustering towards higher intensity values (above 100), as presented in Figure 16. This distribution reflects the consistent availability of natural light during the day, which enhances the overall visibility of traffic signs across a broader intensity range. In contrast, the night-time CDF shows a steeper slope, with intensity values clustered predominantly below 80. This steep curve highlights the significant reduction in sign brightness under night-time conditions, emphasizing the limitations of low retroreflectivity and the challenges associated with artificial illumination. Signs with lower retroreflectivity disproportionately exhibit poor visibility and an inconsistent detection performance.

**Figure 17:**
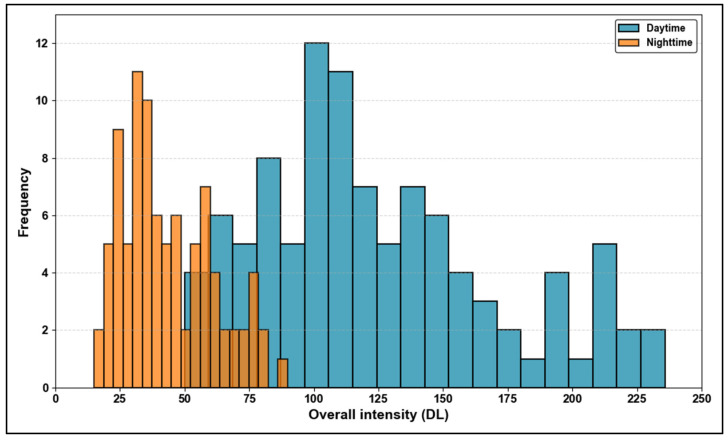
Distribution of overall intensity during daytime and night-time.

The histogram of overall intensity in Figure 17 shows a clear contrast between daytime and night-time conditions. The daytime intensity values are widely distributed, mostly ranging from 50 to 230, reflecting the benefit of natural light for improved sign visibility. In contrast, the night-time intensities cluster between 20 and 80, emphasizing the challenge of maintaining visibility in low-light conditions. The minimal overlap between the two distributions highlights the significant reduction in intensity at night, underscoring the importance of retroreflective materials for reliable night-time visibility.

The analysis of overall intensity reveals that the daytime intensity values are substantially higher and more widely distributed than the night-time values, as evidenced by the statistical metrics and the CDF in Figure 16. The daytime mean intensity of 128 benefits from natural lighting, enhancing visibility and ensuring a reliable detection performance. In contrast, the night-time mean intensity of 42 reflects the challenges posed by reduced light conditions, where the effectiveness of traffic signs relies heavily on their retroreflective properties. The higher variability in night-time intensities (RSD = 45%) further underscores the importance of high-quality retroreflective materials to ensure consistent brightness and visibility. These findings highlight the need for targeted improvements in traffic sign materials, particularly for night-time conditions, to maintain an optimal detection performance and ensure traffic safety under all lighting scenarios.

### 4.4. Sharpness

Sharpness is a crucial metric that reflects the clarity and definition of traffic signs in images, directly influencing their detectability by both human drivers and automated detection systems. Higher sharpness values indicate more defined and distinct traffic signs, making them easier to recognize. In the context of traffic sign images, sharpness is typically measured by analyzing pixel intensity gradients, with higher gradients indicating more distinct edges and details. Sharpness can be influenced by factors such as the quality of the image, lighting conditions, camera settings, and retroreflective properties of the traffic sign material. More apparent, more sharply defined traffic signs are generally better detected under various lighting conditions.

The statistical analysis of sharpness for daytime and night-time conditions reveals significant differences in the clarity of traffic signs under these two lighting environments, as shown in Table 6.

- Daytime Sharpness: The mean sharpness during the day is 8071, with a STD of 5504, resulting in an RSD of 68%. This high variability indicates that while most traffic signs are relatively sharp, there is a considerable fluctuation in sharpness values, likely due to factors such as different sign qualities, the image resolution, and the effects of ambient light.
- Night-time Sharpness: In contrast, the mean sharpness at night is 1452, with an STD of 2284 and a significantly higher RSD of 157%. The much higher RSD at night reflects the large variability in sharpness, where some signs may appear blurred or poorly defined due to factors such as lower retroreflectivity, glare from artificial lighting, or shadows that impact visibility. The stark reduction in mean sharpness at night compared to daytime further highlights the challenges posed by low-light conditions.

### 4.5. Relationship Between On-Site and Camera-Based Human Evaluation Data

**Figure 18:**
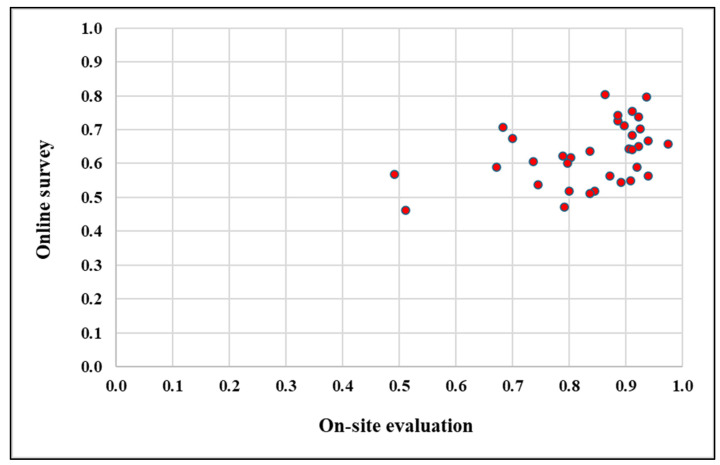
Scatter diagram of on-site vs. online human evaluation scores.

The relationship between the on-site and online evaluation of traffic signs, as shown in Figure 18, indicates a weak correlation between the two methods. The scatter diagram reveals that the scores from the on-site evaluation span a broader range, from 0.49 to 0.98, compared to the narrower range of 0.46 to 0.80 in the online survey. The on-site scores are generally higher, particularly in the middle and upper range of the scale. For instance, when the on-site scores fall within the range of 0.70 to 0.90, the corresponding online scores often remain below 0.80, suggesting that the evaluation conditions significantly influence the results.

Evidence of this disparity appears in the clustering of higher scores. The on-site evaluations frequently produced ratings exceeding 0.90, whereas the online survey rarely exceeded this threshold. Conversely, when the on-site scores were in the lower range (e.g., below 0.60), the online scores were more closely aligned, indicating the consistent underperformance of online scores relative to their on-site counterparts across most ranges.

This pattern underscores the impact of contextual and environmental factors inherent in the on-site evaluations. Factors such as lighting, viewing angle, and the dynamic context of real-world interactions likely contribute to the broader range and generally higher scores in on-site assessments. In contrast, the absence of these factors in the static-image-based online survey restricts participants’ ability to fully evaluate traffic sign visibility, leading to more conservative scores. These findings highlight the limitations of online surveys and suggest that on-site evaluations provide a more comprehensive and favorable representation of traffic sign visibility and effectiveness.

**Figure 19:**
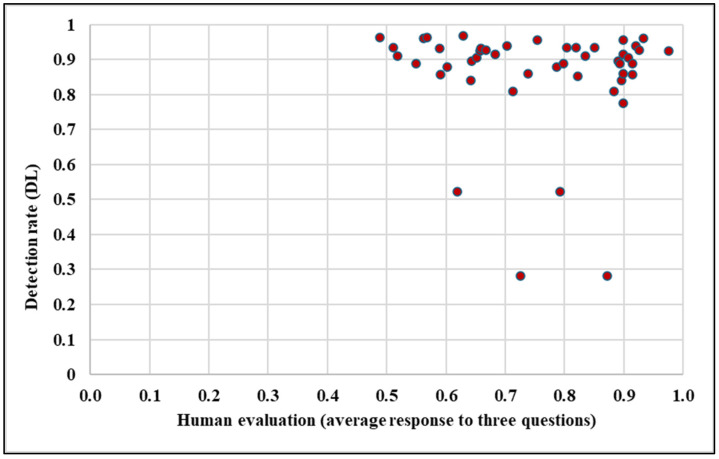
Relation between human evaluations and the detection rate.

The analysis of the relationship between human evaluation scores and the detection rates during daytime, as shown in Figure 19, reveals no significant correlation. While there are instances where higher human evaluation scores align with better detection rates, the overall trend does not consistently support a clear relationship between the two metrics. The mean human evaluation score (0.75) is slightly lower than the mean detection rate (0.88), indicating that the camera system often performs better in specific scenarios. This is especially true for signs with high retroreflective properties or optimal positioning, where the camera system can detect signs more reliably than human evaluators.

Generally, when the average response to the three questions in human evaluation exceeds 0.5, the detection rate tends to exceed 0.8, as illustrated in Figure 19. This implies that higher human evaluation scores are generally associated with a better detection performance, reflecting the system’s effectiveness when rated more favorably by human evaluators.

However, there are notable exceptions to this trend. Sometimes, even when on-site human evaluation scores surpass 0.5, the detection rate does not meet the expected threshold of 0.8. These anomalies suggest that while human evaluation provides valuable insights into the system’s effectiveness, other factors, such as environmental conditions, camera quality, or specific scenarios where the detection model may not perform optimally, influence the detection performance.

However, there are notable exceptions to this trend. Sometimes, even when the on-site human evaluation scores surpass 0.5, the detection rate does not meet the expected threshold of 0.8. These anomalies suggest that while human evaluation provides valuable insights into the system’s effectiveness, other factors, such as environmental conditions, camera quality, or specific scenarios where the detection model may not perform optimally, influence the detection performance.

Considerable variability is observed in the data, as reflected in the RSD values of 16.3% for human evaluation and 15.3% for detection rates. This variability indicates that, in some instances, signs with lower human evaluation scores can still achieve high detection rates. Such discrepancies can be attributed to the camera’s reliance on retroreflectivity and other technical factors that enhance visibility. At the same time, human evaluators may be influenced by subjective visual factors, such as the contrast, angle of observation, or environmental distractions, leading to differences in evaluation outcomes.

An independent *t*-test further revealed a statistically significant difference (*p* < 0.001) between the human evaluation scores and detection rates, emphasizing that they are distinct while the two metrics are related. Human evaluations capture a broader range of subjective visual factors, while camera detection primarily relies on retroreflectivity and algorithmic recognition, which may not always align with human perception.

The findings indicate no clear correlation between human evaluation and the camera-based detection rates. While both metrics emphasize the importance of retroreflective properties and sign visibility, their differing evaluation criteria, with subjective visual impressions versus algorithmic recognition, highlight each assessment method’s distinct roles. Addressing factors such as glare, shadows, and environmental conditions could enhance the performance of both human evaluators and camera systems, ensuring consistent traffic sign detectability. These results underscore the value of using human evaluation as a complementary measure in assessing automated detection systems.

#### Comparison of Human and Algorithmic Methods for Evaluating Traffic Sign

This study employed two complementary methods to assess traffic sign visibility: human evaluations (on-site and online) and algorithmic detection using the YOLOv8 model. Human evaluations provided subjective insights into visibility, readability, and contrast based on real-world perceptions. Viewing angles, environmental distractions, and lighting conditions influenced them. In contrast, the YOLOv8 algorithm relied on objective metrics, such as retroreflectivity and pixel intensity, to evaluate traffic signs.

A key distinction is the ability of the algorithm to maintain consistency across varying conditions, such as low-light scenarios, where human evaluations often exhibit variability due to subjective factors. For instance, human evaluators were more affected by glare or shadows, whereas the algorithm demonstrated robust performance when the retroreflective properties were high. However, in poor retroreflectivity or low-contrast scenarios, human evaluators outperformed the algorithm by relying on contextual understanding and prior knowledge.

These differences highlight the complementary nature of both methods. While human evaluations capture real-world perceptions critical for human-centric design, algorithmic methods excel in providing objective, repeatable assessments, particularly under controlled conditions

### 4.6. Dataset Bias and Model Performance

While diverse and comprehensive, the ZND dataset utilized in this study exhibits certain biases that could influence the model’s performance. A notable bias arises from the overrepresentation of common traffic sign types, such as “Go straight or turn left” and “No entry,” compared to rarer signs like “Bicycle pass” or “Keep left.” This imbalance may lead the model to perform exceptionally well on frequently encountered classes while struggling with underrepresented ones due to limited training samples. Additionally, the dataset contains a higher proportion of images captured during daytime conditions, which may limit the model’s robustness under low-light or night-time scenarios where detection is inherently more challenging. These biases highlight the importance of carefully curating datasets to ensure balanced representation across sign types and environmental conditions, thus improving the generalizability and reliability of traffic sign detection models in real-world applications.

## 5. Conclusions

This study presents a comprehensive framework for traffic sign detection (TSD) and classification (TSC) to address the challenges posed by varying environmental conditions, including lighting disparities between daytime and night-time. By employing the YOLOv8 algorithm, the research demonstrates the efficacy of advanced machine learning techniques in achieving robust detection and classification performance. The novel ZND dataset, comprising 16,500 meticulously labeled images from diverse sources, proved instrumental in training and validating the model. The findings underscore the importance of well-curated datasets in advancing traffic sign recognition technologies and enabling the development of reliable systems for real-world applications.

A key contribution of this work is the integration of retroreflectivity assessments with video analysis, revealing strong correlations between the material properties of signs and their detection performance. Retroreflectivity emerged as a critical determinant of traffic sign visibility, particularly under low-light conditions, emphasizing the need for high-grade materials and regular maintenance to ensure consistent detectability. The analysis also underscored the influence of sharpness, brightness, and contrast on detection accuracy, offering actionable insights into the design and placement of traffic signs.

In addition to algorithmic evaluations, this study incorporated human evaluations to provide a holistic understanding of traffic sign visibility. Human evaluations captured subjective insights influenced by real-world factors, such as viewing angles and environmental distractions, offering critical perspectives for human-centric system design. In contrast, the YOLOv8 model delivered consistent, objective evaluations, excelling in scenarios with high retroreflectivity and image quality. These complementary approaches highlight the value of integrating human perception and algorithmic detection methods to enhance the design and functionality of AV systems. This integration can ensure that AV systems align with human expectations while maintaining objective performance benchmarks, contributing to more human-centric and trustworthy intelligent transportation systems.

The findings of this research have significant implications for the development of intelligent transportation systems and AV technologies. This study recommends enhancing traffic sign detection reliability through improved materials, algorithmic innovations, and standardized retroreflectivity assessments by bridging the gap between theoretical advancements and practical applications. Furthermore, the insights gained can inform the design of next-generation traffic signs and influence policymaking for road safety standards.

This study lays a strong foundation for addressing critical traffic sign recognition and quality assessment gaps, ultimately contributing to safer, more efficient, and human-centric autonomous vehicle systems. This research supports the evolution of intelligent transportation systems and global road safety initiatives by bridging the gap between theoretical advancements and practical applications.

Table 7 compares these methods, highlighting their respective costs, accuracy, limitations, and applications across various environmental conditions. It also emphasizes each method’s strengths and constraints regarding its practical use in traffic sign evaluation.

## 6. Future Work and Recommendations

The findings of this study offer valuable insights into traffic sign detection and classification using the YOLOv8 model. However, there remain several opportunities for further research to address the study’s limitations and explore advanced techniques for improving performance under diverse real-world conditions. Below, future research directions and recommendations are presented in a structured manner.

- Investigate the fusion of LiDAR and camera-based systems to enhance detection accuracy. LiDAR can provide spatial and reflectivity data, while cameras can capture detailed visual and color information, enabling more robust detection in diverse environments.
- Extend the study to include RA3 retroreflective materials to evaluate their impact on detection rates compared to RA1 and RA2. This would provide a more comprehensive understanding of retroreflective effects on traffic sign recognition.
- Conduct systematic experiments with hyperparameter tuning, including varying batch sizes, learning rates, and input image sizes, to identify the optimal configurations for balancing model accuracy and training efficiency.
- Expand the dataset to include more balanced representations of rare traffic sign types and additional nighttime scenarios. This could involve oversampling underrepresented classes and capturing images in low-light and adverse weather conditions.
- To better understand their impact on model performance, perform ablation studies to isolate the contributions of key components, such as data augmentation techniques and specific architectural layers (e.g., CSPNet, PANet).
- Explore advanced image-processing methods, such as adaptive exposure control, low-light image enhancement, and glare reduction, to improve detection performance under challenging conditions like night-time glare or adverse weather.
- Investigate the integration of human perception insights into AV decision-making by analyzing findings from human evaluations. This would help develop more human-centric autonomous vehicle systems by improving interaction, trust, and system safety.
- Test the model under more extreme environmental scenarios, such as heavy rain, dense fog, and snow, to assess its robustness and reliability in real-world autonomous vehicle systems.

By addressing these areas, future research can contribute to developing more accurate, robust, and generalizable traffic sign detection systems, ultimately improving the safety and functionality of autonomous vehicles.

## Figures and Tables

**Table 1:** Exemplar questionnaire for traffic sign evaluation.

No.	Questions					Sign Image
**1**	How clearly visible is the traffic sign?					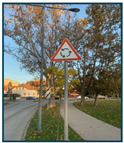
	(1 = Not visible at all, 5 = Very visible)					
	1	2	3	4	5	
**2**	How easily could you read the text and symbols on the traffic sign?					
	(1 = Not readable at all, 5 = Very readable)					
	1	2	3	4	5	Sign No. 3
**3**	How well did the colors of the traffic sign stand out from their surroundings?					
	(1 = Poor contrast, 5 = Excellent contrast)					
	1	2	3	4	5	

**Table 2:** Analysis of the detection rate for traffic signs in daytime and night-time.

Light Condition	No. of Signs	Detection Rate		
		Mean	Standard Deviation	Relative Standard Deviation
**Day**	100	0.82	0.18	22%
**Night**	90	0.83	0.17	20%
**Miss detection at Night**	10			

**Table 3:** Independent samples *t*-test.

	*t*-Test for Equality of Means						
	*t*	df	Sig. (Two-Tailed)	Mean Difference	Std. Error Difference	95% Confidence Interval of the Difference	
						Lower	Upper
**Equal variances assumed**	−0.337	178	0.736	−0.01	0.024	−0.056	0.040

**Table 4:** Statistical comparison of retroreflectivity and detection rates for RA1 and RA2 materials.

Traffic Sign Sheet Class		RA1	RA2
Number of Signs		44	11
**Retroreflectivity coefficients**	Mean	29	187
	Standard Deviation	18.0	89.9
	Relative Standard Deviation	63%	48%
**Detection rate (day)**	Mean	0.80	0.88
	Standard Deviation	0.17	0.05
	Relative Standard Deviation	21%	6%
**Detection rate (night)**	Mean	0.83	0.87
	Standard Deviation	0.19	0.08
	Relative Standard Deviation	23%	9%

**Table 5:** Analysis of overall intensity for traffic signs in daytime and night-time.

Light Condition	No. of Signs	Overall Intensity		
		Mean	Standard Deviation	Relative Standard Deviation
**Day**	100	128	52	41%
**Night**	90	42.3	19	45%

**Table 6:** Analysis of sharpness during daytime and night-time.

Light Condition	No. of Signs	Sharpness		
		Mean	Standard Deviation	Relative Standard Deviation
**Day**	100	8071	5504	68%
**Night**	90	1452	2284	157%

**Table 7:** Comparison of traffic sign evaluation methods.

Criteria	Methods		
	Handheld Retroreflectometer	Camera	Human Evaluation
**Cost**	Low	Medium	Low
**Measuring Accuracy**	High (for retroreflectivity)	Moderate (dependent on image quality)	Low to Moderate (subjective)
**Performance in Low Light**	High	Decreased (glare, shadows)	High (captures real-world conditions)
**Performance in Poor Weather**	Performs well (unaffected by weather)	Affected (e.g., rain, fog)	Dependent on the evaluator’s experience and environmental conditions
**Data Interpretation Complexity**	Low (simple direct measurements)	High (requires advanced algorithms)	Very low (subjective, perception-based)
**Advantages**	Accurate, objective measurement of retroreflectivity.	Captures environmental factors (lighting, placement).	Captures subjective, real-world insights about sign visibility.
	Simple to use in field settings.	Suitable for traffic sign recognition and classification.	Reflects road user perception.
**Limitations**	Limited to retroreflectivity measurements.	Sensitive to environmental conditions (glare, shadows).	Subject to variability across evaluators.
	It cannot capture environmental or contextual factors.	Requires substantial computational resources.	Inconsistent and subjective, not standardized.
**Applications**	Retroreflectivity measurement of traffic signs.	Analyzing contextual visibility factors.	Assessing perceived visibility and clarity of traffic signs in real-world settings.
	Monitoring compliance with retroreflectivity standards.	Traffic sign recognition and classification.	

## Data Availability

The ZND dataset used in our study is freely available online and can be accessed at: https://www.kaggle.com/datasets/endaziar/znd-dataset (accessed on 2 September 2024).

## Abbreviations

The following abbreviations are used in this manuscript:

RA	Retroreflectivity coefficient
AV	Autonomous vehicle
YOLO	You Only Look Once
DL	Deep learning
TSD	Traffic sign detection
TSC	Traffic sign classification
ADAS	Advanced driver assistance systems
GTSRB	German traffic sign recognition benchmark
